# Supplementary figures and images for: Multinomial Convolutions for Joint Modeling of Regulatory Motifs and Sequence Activity Readouts
Source: Genes (Basel). 2022 Sep 8;13(9):1614. doi: 10.3390/genes13091614 (PMC9498894; doi:10.3390/genes13091614)

(A) Novel Repressors

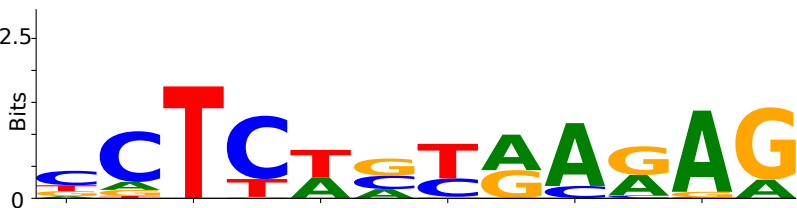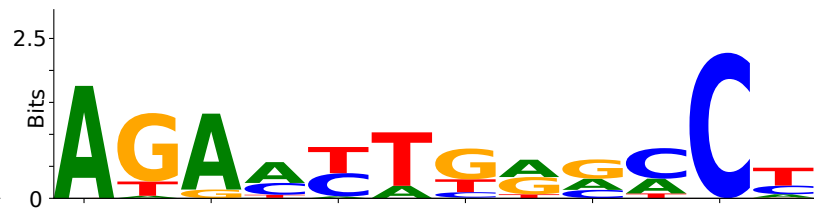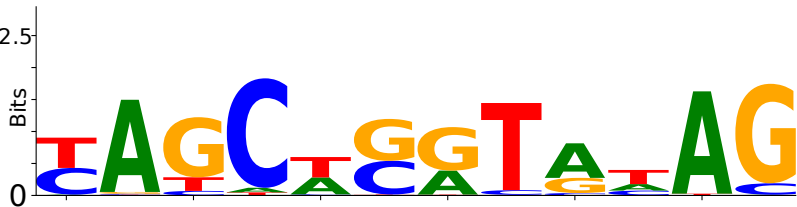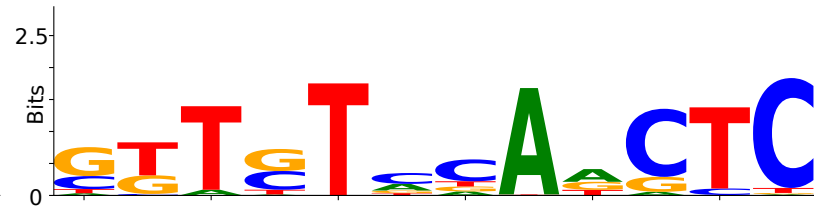

(B) Novel Activator

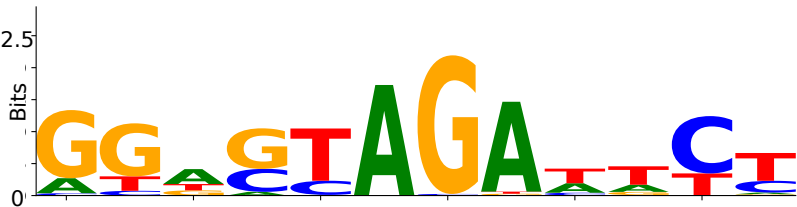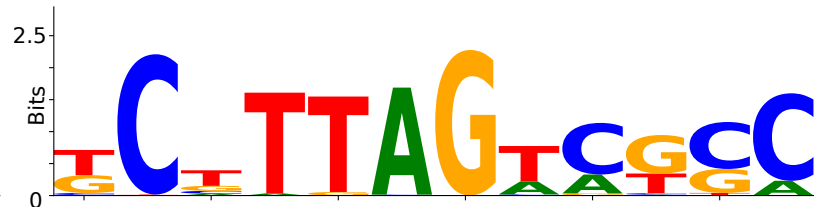

Supplement: Supplementary file 1 [file genes-13-01614-s001.zip › genes-1866857-supplementary.pdf]
